# Opening the Dialogue on Death: The Value of Intergenerational Death Cafés

**DOI:** 10.1093/geroni/igaf122.876

**Published:** 2025-12-31

**Authors:** Tamar Shovali

**Affiliations:** Eckerd College, St. Petersburg, Florida, United States

## Abstract

This project intentionally fostered open, honest conversations about mortality by building an intergenerational Death Café, recognizing that such spaces are not inherently intergenerational. Undergraduates in a Death and Dying course, community members, and members of our university-affiliated Academy of Senior Professionals joined a series of Death Cafés in 2023 and 2024, with an average of 43 participants ranging in age from 18 to 84 years. Attendees were surveyed following each session about their frequency of engagement in discussions about death and dying in their daily lives, their perceptions of the value of intergenerational Death Cafés, whether they learned something new about death and dying, and whether their views on death and dying changed as a result of attending. Average responses indicate consistently high value and learning across all sessions, with a 12.2% increase in participants responding affirmatively that their views on death and dying changed. Nearly half of attendees reported rarely discussing death and dying in their lives, though such conversations increased slightly from 33.3% after the first café to 58% after the third café. Qualitative results indicate that each café built upon the last, shifting from initial openness and comfort (café 1), to refining the dialogue process and balancing perspectives (café 2), to encouraging deeper personal action and longer-term comfort with the subject (café 3). Clearly, meaningful intergenerational conversations about mortality can foster fulfilling and deeply human connections. The process of preparing and planning cafés will be discussed in this presentation.

